# Sex differences in body mass index and waist circumference trajectories and dementia risk: the HUNT4 70+ study

**DOI:** 10.1007/s11357-025-01660-3

**Published:** 2025-04-22

**Authors:** Ekaterina Zotcheva, Bjørn Heine Strand, Vegard Skirbekk, Kay Deckers, Steinar Krokstad, Gill Livingston, Archana Singh-Manoux, Geir Selbæk

**Affiliations:** 1 Norwegian National Centre for Ageing and Health, Vestfold Hospital Trust, Aldring Og Helse, PO Box 2136, 3103 Tønsberg, Norway; 2 Department of Geriatric Medicine, Oslo University Hospital, Nydalen, OUS HF, Ullevål Sykehus, PO Box 4956, 0424 Oslo, Norway; 3 Department of Physical Health and Ageing, Norwegian Institute of Public Health, Skøyen, PO Box 222, 0213 Oslo, Norway; 4 Centre for Fertility and Health, Norwegian Institute of Public Health, PO Box 222, 0213 SkøyenOslo, Norway; 5 Department of Psychiatry and Neuropsychology, Mental Health and Neuroscience Research Institute (MHeNs), Alzheimer Centrum Limburg, Maastricht University, PO Box 616, 6200 MD Maastricht, The Netherlands; 6 Department of Public Health and Nursing, Faculty of Medicine and Health Sciences, HUNT Research Centre, Norwegian University of Science and Technology, PO Box 8905, 7491 Trondheim, Norway; 7 Levanger Hospital, Nord-Trøndelag Hospital Trust, PO Box 333, 7601 Levanger, Norway; 8 Division of Psychiatry, University College London, 149 Tottenham Ct Rd, London, W1 T7 NF UK; 9 North London NHS Foundation Trust, 4 St Pancras Way, London, NW1 OPE UK; 10 Epidemiology of Ageing and Neurodegenerative Diseases, U1153 Inserm, Université Paris Cité, 10 Avenue de Villemin, 75010 Paris, France; 11 Institute of Clinical Medicine, Faculty of Medicine, University of Oslo, Blindern, PO Box 1072, 0316 Oslo, Norway

**Keywords:** Anthropometric measures, Cognitive impairment, Life-course perspective, Adiposity, Population-based study

## Abstract

We examined associations between body mass index (BMI), waist circumference (WC), and dementia risk, and differences in BMI and WC trajectories before dementia diagnosis. We included 9,739 participants (54% women) aged 70+ from the Trøndelag Health Study (HUNT4 70+). BMI was measured four times (1984–2019) and WC three times (1995–2019). Dementia diagnoses were clinically assessed at HUNT4 70+ . Women and men with dementia had higher midlife BMI and WC than those without dementia. These differences diminished closer to diagnosis, especially in women. Midlife obesity in both sexes and midlife overweight, high WC, and overweight/obesity with high WC in men were linked to higher dementia risk. Lower dementia risk was observed with late-life overweight for both sexes, late-life high WC in women, late-life overweight/obesity with normal WC in men or high WC in women. Adiposity measures and their changes influence dementia risk differently in women and men.

**Supplementary Information:**

The online version contains supplementary material available at 10.1007/s11357-025-01660-3.

## Introduction

Recent predictions indicate that global dementia prevalence will triple within the next 30 years [1]. Alongside therapeutic options, there is considerable emphasis on dementia prevention, focusing on modifiable risk and protective factors. The Lancet Commission on Dementia Prevention, Intervention, and Care has identified 14 such factors, potentially accounting for as much as 45% of dementia cases [2]. Among these, midlife obesity (body mass index [BMI] > 30 kg/m^2^) is highlighted as a key risk factor, though research on the link between obesity and dementia remains inconclusive [3].

Meta-analyses suggest that both midlife obesity and underweight (BMI < 18.5 kg/m^2^) are associated with higher dementia risk, with no such link in midlife overweight (BMI > 25 kg/m^2^) [4, 5]. Similarly, a study involving 2.8 million adults showed that underweight individuals and those with annual weight loss > 0.5% had elevated dementia risks, whereas those with an upper-normal BMI (22.5–24.9 kg/m^2^) had lower risk [6]. While midlife obesity has consistently been linked to a higher dementia risk, obesity after age 65 appears to be protective, likely due to weight loss driven by preclinical disease [5, 7, 8].

Despite its widespread use, BMI does not account for variations in body fat distribution [9] that may better explain obesity-related health risks [10–12]. Central adiposity, often measured by waist circumference (WC), is an alternative indicator, but results in relation to dementia are mixed. A meta-analysis of over 5 million individuals found higher WC after, but not before, age 65 to be associated with higher dementia risk [13]. A Korean study with more than 850,000 participants linked high WC in adults ≥ 65 years to higher dementia risk, even in individuals with normal BMI [14], highlighting the distinct contribution of central adiposity. Conversely, results from the Whitehall II Study showed higher dementia risk among individuals with obesity or high WC at age 50, but not at ages 60 or 70 [15]. A recent study in older adults (mean age 71) found that a 1-year reduction in BMI was associated with a higher risk of Alzheimer’s disease in participants without obesity, whereas WC reduction was linked to a higher risk only in participants with obesity [16]. These findings highlight the importance of considering both BMI and WC in dementia research.

Dementia prevalence is higher in women than men [17, 18], though the mechanisms underlying this disparity remain unclear. Few studies have explored sex differences in anthropometric measures and dementia risk, with mixed results. One study of individuals aged 45–74 followed for 8 years reported higher dementia risk in overweight women but a lower risk in men, and higher risk with low WC in both sexes [19]. A longitudinal study in 296,767 participants aged ≥ 65 followed for up to 10 years found no link between obesity and dementia, but overweight women had lower dementia risk and underweight men had higher dementia risk [20]. These studies were limited by follow-up periods of up to 10 years, did not track changes in BMI or WC, and only one study used measures of central adiposity [19], underscoring the need for further investigation.

The inconsistencies in existing research highlight the need for robust studies tracking changes in BMI and WC over time in both sexes, particularly from midlife to older age. Few studies have examined the effects of both BMI and WC at different ages on dementia risk, which may offer a more nuanced understanding of how weight and fat distribution interact to influence cognitive outcomes. To address these gaps, we utilized data from a large Norwegian population-based study, tracking BMI and WC up to 35 years, with a clinical cognitive evaluation of all participants at age 70 and older. Our primary objective was to examine BMI and WC trajectories throughout adulthood in relation to dementia risk at age 70 and older, and to assess potential sex differences. We also aimed to explore the joint effects of BMI and WC on dementia risk.

## Methods

### Study population

**Fig. 1:**
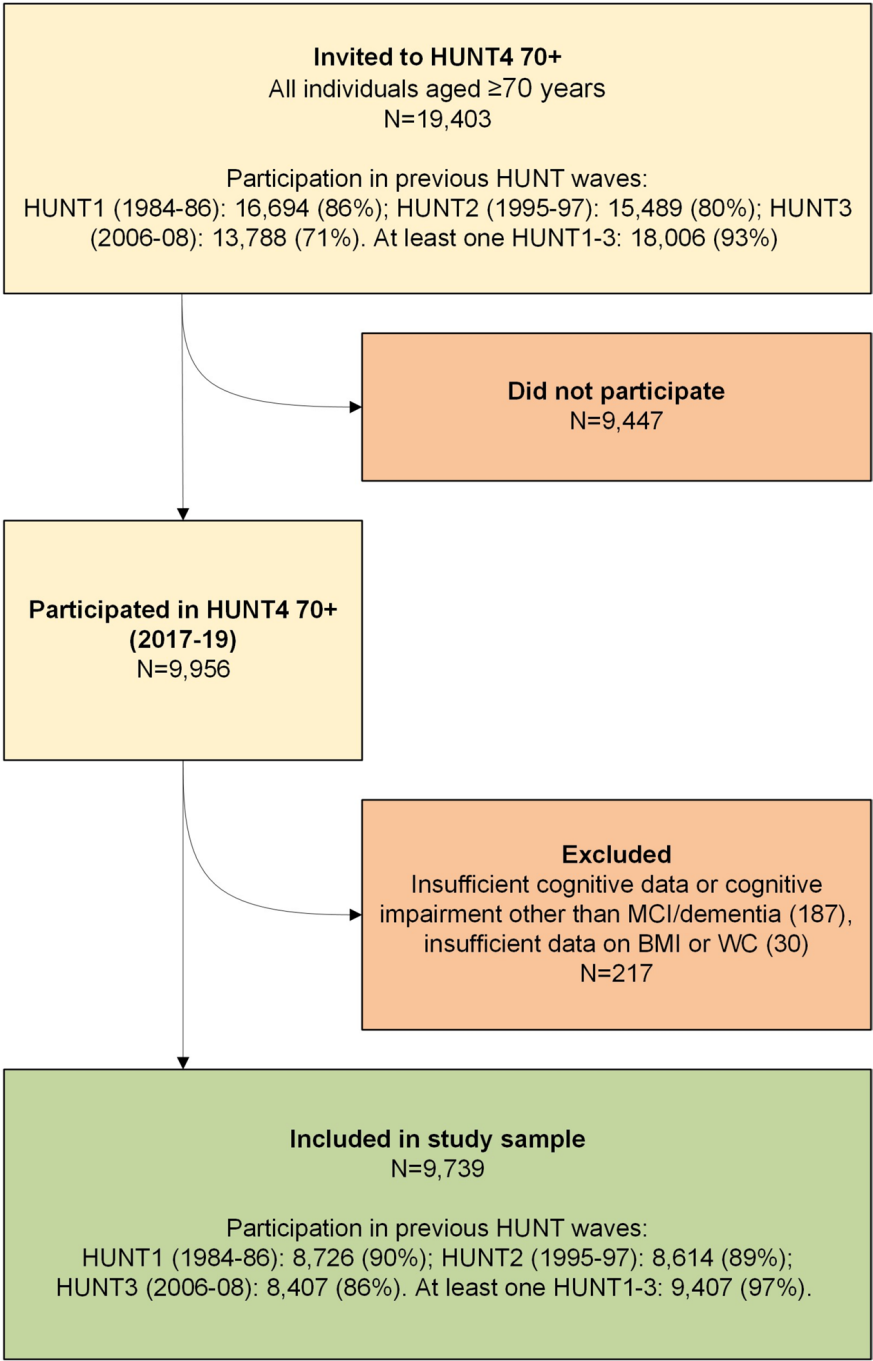
Flowchart showing study sample selection from the Trøndelag Health Study (HUNT)

The Trøndelag Health Study (HUNT) is an extensive ongoing study conducted in Trøndelag County, Norway. All adult inhabitants of the northern county area were invited to four consecutive study waves consisting of questionnaires and clinical measurements: HUNT1 (1984–86), HUNT2 (1995–97), HUNT3 (2006–08), and HUNT4 (2017–19). The study sample for the present analyses was drawn from those participating in the 70+ sub-study at the fourth wave of the HUNT Study (HUNT4 70+, conducted from 2017 to 2019). During HUNT4, all residents of the northern county are aged ≥ 70 years (*n* = 19,403) were invited to take part in the 70+ sub-study, and 9,956 (51.3%) individuals aged 70–104 participated. Further details on the HUNT Study are provided elsewhere [18, 21, 22]. We excluded HUNT4 70+ participants who had incomplete cognitive data or who had causes of cognitive impairment other than dementia or mild cognitive impairment (MCI) (*n* = 187) and participants with missing data on BMI or WC across all of the HUNT waves (*n* = 30). The final analytical sample comprised 9,739 participants (5,299 women and 4,440 men) from HUNT4 70 + (Fig. 1).

### Ethics

All participants gave informed consent regarding the use of data from questionnaires, biological samples, and linkage to national registries for research purposes upon participation. For participants without the capacity to consent, a next-of-kin gave consent on behalf of the participant. This procedure was approved by the Norwegian Data Protection Authority and the Regional Ethics Committee in Norway. The present study was approved by the Regional Ethics Committee in Norway (REK Sør-Øst A, 645862) and the HUNT board of directors.

### Body mass index and waist circumference

BMI and WC were measured by trained staff using standardized protocols at the clinical examinations in the HUNT Study. Body weight (to the nearest half kilogram [kg]) and height (in whole centimeters [cm]) was measured with the participants wearing light clothing and no shoes. BMI was calculated by dividing body weight (in kg) by height in meters squared (kg/m^2^). BMI was measured four times; at HUNT1, 2, 3, and 4, providing a maximum span of 35 years (1984–2019). WC was measured at the height of the umbilicus to the nearest whole cm with the person in a standing position with arms hanging relaxed. WC was measured three times; at HUNT2, 3, and 4, providing a maximum span of 24 years (1995–2019) of data on WC.

BMI was used both as a continuous variable (in kg/m^2^), and as a categorical variable according to the standard categorization of BMI: underweight (< 18.5 kg/m^2^), normal weight (18.5–24.9 kg/m^2^), overweight (25–29.9 kg/m^2^), and obesity (≥ 30 kg/m^2^) [23]. WC was used as both a continuous variable (in cm), and as a categorical variable according to sex-specific cut-offs [24]: normal WC (women: ≤ 88 cm, men: ≤ 102 cm), high WC (women: > 88 cm, men: > 102 cm).

To examine joint associations of BMI and WC with dementia risk, we created four combination groups of BMI and WC at the three HUNT waves where both BMI and WC were available (HUNT2-4). To increase statistical power, participants with overweight or obesity were grouped together, whereas participants with underweight were excluded from these analyses due to small numbers in this group (HUNT2: *n* = 26; HUNT3: *n* = 30; HUNT4: *n* = 95). For these analyses, the following four combination groups were created: normal WC normal BMI, normal WC overweight/obesity, high WC normal BMI, and high WC overweight/obesity.

### Dementia

In HUNT4 70+, all participants underwent clinical assessments for dementia and MCI. This evaluation encompassed cognitive function, activities of daily living, neuropsychiatric symptoms, disease progression, and interviews with family caregivers. Two specialists from a pool of nine experts in geriatrics, neurology, or old age psychiatry used all available data to classify cognitive impairment following DSM-5 diagnostic criteria [25]. Based on these assessments, participants were classified into three categories: no cognitive impairment, MCI (mild neurocognitive disorder), and dementia (major neurocognitive disorder). Details on the diagnostic process have been published elsewhere [18]. In the current study, participants’ cognitive status was dichotomized into no dementia (no cognitive impairment or MCI) or dementia.

### Other covariates

We included several potential confounders in the association between BMI or WC trajectories and dementia. The selection of potential confounders was done a priori based on existing literature and a directed acyclic graph (DAG) provided in the Supplementary materials (Fig S1). Data on potential confounders were obtained from the questionnaires and clinical measurements at HUNT1-4. We included the following time invariant covariates: sex (woman/man), educational attainment (primary/secondary/tertiary), marital status (unmarried or divorced/married/widow[er]), and APOE ɛ4 carrier (no ɛ4 allele/ɛ4-heterozygote/ɛ4-homozygote). In addition, the following time-dependent covariates from all four HUNT waves were included: age, physical activity (1 h or more weekly/less than 1 h weekly), smoking (never or former daily smoker/daily smoker), and symptoms of anxiety or depression (no/yes). We chose not to include variables such as diabetes, hypertension, and LDL cholesterol as adjusting for these conditions would likely obscure mediating pathways between BMI/WC and dementia, given that these conditions are known potential consequences of obesity [26, 27] and established risk factors for dementia [2]. A more thorough definition of the potential confounders is provided in the Supplementary materials.

### Statistical analysis

All analyses were performed in Stata 18.0. Inverse-probability weighting (IPW) was used to account for non-response. The probability of participation was calculated using logistic regression including registry-based data on age, sex, and education available on those invited to HUNT4 70+ (*n* = 19,463, of whom 9,956 participated). As the IPW is based on education and the weights were applied to the data set prior to further imputation, missing data on education (*n* = 66) were manually imputed as primary education. To handle missing values on other potential confounders (Table S1), we performed multiple imputation by a chained equation including all non-missing study variables, producing twenty imputed data sets. The results from the imputed datasets were combined using Rubin's rules to ensure valid statistical inferences. The proportion of missing data for each potential confounder is provided in the Supplementary materials (Table S1). A *P*-value < 0.05 was considered an indicator of statistical significance.

We performed multilevel mixed-effects linear regression models with random intercept and random slope to investigate longitudinal trajectories of BMI and WC across the HUNT waves among participants with and without a dementia diagnosis at HUNT4. These trajectories were modeled using a backward timescale, meaning that the dementia assessment at HUNT4 served as time 0. BMI and WC at each HUNT wave (HUNT1-4 for BMI, HUNT 2–4 for WC) served as outcome variables, and dementia status (no dementia vs dementia) at HUNT4, time (coded as HUNT1-4), and potential confounders as independent variables. An interaction term between time and dementia status (time x dementia) was included in the model. This method allowed us to test for differences in the trajectories of BMI and WC between individuals with and without dementia at HUNT4. Potential sex differences were examined by including an interaction term between time, dementia, and sex (time x dementia x sex) in the model, and all analyses were thereafter performed stratified by sex. The regression models were performed in two steps: model 1: adjusted for age, and model 2: the fully adjusted model had additional adjustments for educational attainment, marital status, physical activity, smoking, symptoms of anxiety or depression, and presence of the APOE ɛ4 allele. Absolute differences in mean BMI and WC, presented as mean differences (*MD*), were predicted post hoc based on dementia status and HUNT wave using the margins command.

We modelled potential non-linear associations between BMI and WC at the four HUNT waves and dementia risk using fully adjusted logistic regression with restricted cubic splines. We generated spline variables for BMI and WC at each HUNT wave, fitted logistic regression models with interaction terms between the spline variables and sex, and predicted the probabilities of dementia at HUNT4 for women and men. The associations were visualized using the marginsplot command.

To estimate the association between categories of BMI and WC assessed at the four HUNT waves with dementia risk at HUNT4, we used multiple logistic regression models to calculate odds ratios (OR) with 95% confidence intervals (CI) for dementia. Categorical BMI (normal vs. underweight, overweight, and obesity) at HUNT1-4 and WC (normal vs. high) at HUNT2-4 served as exposure variables in separate models for each HUNT wave and each exposure variable with dementia at HUNT4 as outcome. The logistic regression models were performed in one step, adjusting for all variables in model 2 as described above. Further, to investigate the joint effect of BMI and WC on dementia risk, we used combination groups of BMI and WC at HUNT2-4 to predict dementia risk at HUNT4 using logistic regression models adjusted for all variables in model 2 (see above). An interaction term between BMI, WC, or combination groups of BMI and WC and sex (BMI x sex or WC x sex or BMI/WC combination x sex) was included in the logistic regression models, and all models were run separately for women and men.

Finally, to check the robustness of our findings, we performed two sensitivity analyses. First, we repeated the multilevel mixed-effects linear regression and logistic regression analyses in participants with complete data on all potential confounders (*n* = 8,450). Second, as participants with MCI were grouped together with participants without cognitive impairment in the main analyses, we repeated the multilevel mixed-effects linear regression and logistic regression analyses after removing participants with MCI (*n* = 3,412) from the study sample. Here, we repeated the previously described modelling steps, including multiple imputation of missing potential confounders.

## Results

In our study sample (*n* = 9,739, 54% women), the prevalence of dementia at age 70 years and older was 1,507 (15.5%); 890 (16.8%) in women and 617 (13.9%) in men. All participants in the study sample had at least one BMI measurement, and 9,608 participants (99%) had at least one WC measurement during the study period. There was significant interaction between time, dementia, and sex on both WC and BMI in the multilevel mixed-effects linear regression, between sex and categories of BMI at HUNT1 and HUNT2 on dementia, between sex and categories of WC at HUNT2, HUNT3, and HUNT4 on dementia, and between sex and combination groups of BMI and WC at HUNT2 and HUNT4 on dementia (all *P* < 0.05). Time-dependent characteristics of women and men in the study sample are displayed in Table 1, whereas time invariant characteristics are presented in Table 2. Characteristics of the entire study sample (not stratified by sex) are provided in the Supplementary materials (Tables S2 and S3).

**Table 1:** Time-dependent characteristics of study sample (*n* = 9,739) at the four HUNT Study waves for women and men with BMI and/or WC data at each wave, stratified by dementia status at HUNT4

	No dementia at HUNT4				Dementia at HUNT4			
	HUNT1 (1984–86)	HUNT2 (1995–97)	HUNT3 (2006–08)	HUNT4 (2017–19)	HUNT1 (1984–86)	HUNT2 (1995–97)	HUNT3 (2006–08)	HUNT4 (2017–19)
Women, N in sample (%)	4,409 (83.2)				890 (16.8)			
N with BMI and/or WC data (%)	3,993 (90.6)	3,981 (90.3)	3,924 (89.0)	4,348 (98.6)	812 (91.2)	792 (89.0)	673 (75.6)	687 (77.2)
Age, mean (SD)	44.5 (5.9)	55.5 (5.9)	66.3 (5.7)	77.3 (5.7)	52.1 (7.4)	63.1 (7.4)	73.5 (7.1)	84.1 (7.2)
< 1 h weekly physical activity*, *n* (%)	877 (22.0)	2,947 (74.0)	2,313 (58.9)	2,331 (53.6)	194 (23.9)	491 (62.0)	339 (50.4)	144 (21.0)
Daily smoker*, *n* (%)	856 (21.4)	837 (21.0)	459 (11.7)	299 (6.9)	180 (22.2)	161 (20.3)	72 (10.7)	35 (5.1)
Symptoms of anxiety or depression*, *n* (%)	433 (11.1)	697 (17.5)	638 (16.3)	673 (15.5)	119 (14.7)	165 (20.8)	150 (22.3)	148 (21.5)
BMI, mean (SD)	24.3 (3.5)	26.7 (4.1)	27.7 (4.5)	27.2 (4.8)	25.5 (4.1)	27.5 (4.5)	27.9 (4.8)	26.5 (5.2)
Underweight, *n* (%)	37 (0.9)	13 (0.3)	17 (0.4)	56 (1.3)	5 (0.6)	7 (0.9)	8 (1.2)	24 (3.5)
Normal BMI, *n* (%)	2,595 (65.0)	1,468 (36.9)	1,114 (28.4)	1,417 (32.6)	407 (50.1)	237 (29.9)	182 (27.0)	268 (39.0)
Overweight, *n* (%)	1,085 (27.2)	1,782 (44.8)	1,742 (44.4)	1,788 (41.1)	204 (37.4)	361 (45.6)	279 (41.5)	241 (35.1)
Obesity, *n* (%)	276 (6.9)	717 (18.0)	1,050 (26.8)	1,085 (25.0)	96 (11.8)	186 (23.5)	200 (29.7)	153 (22.3)
WC, mean (SD)	N/A	82.1 (10.4)	92.6 (12.0)	94.3 (12.6)	N/A	84.3 (11.0)	93.1 (12.4)	90.6 (13.1)
High WC*, *n* (%)	N/A	962 (24.2)	2,398 (61.1)	2,658 (61.1)	N/A	262 (33.1)	431 (64.0)	143 (20.8)
Men, N in sample (%)	3,823 (86.1)				617 (13.9)			
N with BMI and/or WC data (%)	3,311 (86.6)	3,292 (86.1)	3,320 (86.8)	3,785 (99.0)	548 (88.8)	525 (85.1)	470 (76.2)	499 (80.9)
Age, mean (SD)	43.9 (5.5)	55.0 (5.5)	66.0 (5.5)	76.8 (5.4)	49.3 (7.2)	60.4 (7.3)	71.0 (6.9)	81.4 (6.9)
< 1 h weekly physical activity*, *n* (%)	908 (27.4)	2,484 (75.5)	1,860 (56.0)	2,249 (59.4)	142 (25.9)	339 (64.6)	230 (48.9)	169 (33.9)
Daily smoker*, *n* (%)	738 (22.3)	664 (20.2)	353 (10.6)	190 (5.0)	132 (24.1)	107 (20.4)	49 (10.4)	29 (5.8)
Symptoms of anxiety or depression*, *n* (%)	280 (8.5)	556 (16.9)	407 (12.3)	502 (13.3)	55 (10.0)	91 (17.3)	81 (17.2)	94 (18.8)
BMI, mean (SD)	25.1 (2.6)	26.7 (3.0)	27.7 (3.4)	27.4 (3.8)	25.6 (2.9)	27.1 (3.3)	27.6 (3.4)	26.8 (4.2)
Underweight, *n* (%)	3 (0.1)	4 (0.1)	5 (0.2)	11 (0.3)	3 (0.6)	2 (0.4)	0 (0.0)	4 (0.8)
Normal BMI, *n* (%)	1,699 (51.3)	926 (28.1)	656 (19.8)	1,002 (26.5)	233 (42.5)	126 (24.0)	107 (22.8)	164 (32.9)
Overweight, *n* (%)	1,447 (43.7)	1,938 (58.9)	1,933 (58.2)	1,966 (51.9)	275 (50.2)	308 (58.7)	256 (54.5)	234 (46.9)
Obesity, *n* (%)	162 (4.9)	423 (12.9)	720 (21.7)	804 (21.2)	37 (6.8)	89 (17.0)	105 (22.3)	95 (19.0)
WC, mean (SD)	N/A	92.4 (7.9)	98.8 (9.5)	100.4 (12.8)	N/A	94.0 (8.9)	99.8 (9.4)	100.4 (13.7)
High WC*, *n* (%)	N/A	314 (9.5)	1,041 (31.4)	1,438 (38.0)	N/A	92 (17.5)	166 (35.3)	127 (25.5)

*APOE* apolipoprotein E, *BMI* body mass index (kg/m^2^), *HUNT* The Trøndelag Health Study, *SD* standard deviation, *WC* waist circumference (cm). *High WC: women > 88 cm, men > 102 cm

**Table 2:** Time invariant characteristics of women and men in the study sample, stratified by dementia status at HUNT4

	No dementia	Dementia
Women		
*N* (%)	4,409 (83.2)	890 (16.8)
Education*, *n* (%)		
Primary	2,420 (54.9)	648 (72.8)
Secondary	1,221 (27.7)	179 (20.1)
Tertiary	768 (17.4)	63 (7.1)
Married**, *n* (%)	3,615 (82.0)	709 (80.0)
APOE ɛ4 presence, *n* (%)	1,202 (27.3)	353 (39.7)
Men		
*N* (%)	3,823 (86.1)	617 (13.9)
Education*, *n* (%)		
Primary	1,484 (38.8)	348 (56.4)
Secondary	1,334 (34.9)	206 (33.4)
Tertiary	1,005 (26.3)	63 (10.2)
Married^†^, *n* (%)	3,000 (78.5)	388 (79.1)
APOE ɛ4 presence, *n* (%)	1,145 (30.0)	210 (34.0)

*At HUNT1, HUNT2, and/or HUNT4. ^†^At HUNT1. Abbreviations: APOE: apolipoprotein E, HUNT: The Trøndelag Health Study

### BMI trajectories

**Fig. 2:**
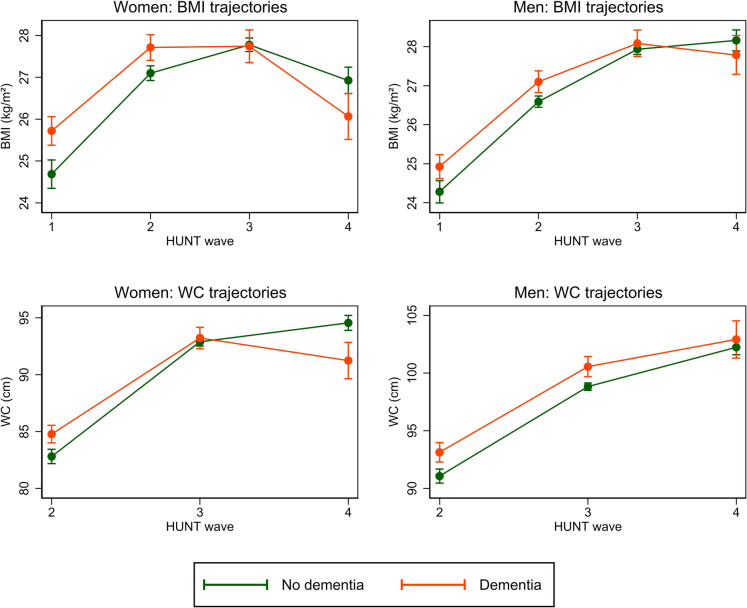
Trajectories of body mass index (BMI) from HUNT1 to HUNT4 and trajectories of waist circumference (WC) from HUNT2 to HUNT4 in women and men according to dementia status at HUNT4. Adjusted for age, educational attainment, marital status, physical activity, smoking, symptoms of anxiety or depression, and APOE ɛ4 status. Data on adjustment variables are imputed using multiple imputation. Abbreviations: HUNT: The Trøndelag Health Study, HUNT1: 1984–86, HUNT2: 1995–97, HUNT3: 2006–08, HUNT4: 2017–19

Trajectories of BMI from HUNT1 to HUNT4 among participants with and without dementia differed between women and men (Table 3, Fig. 2). Both women and men with dementia had higher BMI at HUNT1 and HUNT2 compared to women and men without dementia, with larger differences in women. At HUNT3, women and men with dementia had approximately the same BMI as women and men without dementia. At HUNT4, however, women with dementia had lower BMI compared to women without dementia, whereas the difference in BMI of men with and without dementia was smaller and not statistically significant (Table 3, Fig. 2).

**Table 3:** Absolute difference in mean body mass index (BMI) presented as mean difference (*MD*) with 95% CI at HUNT1, HUNT2, HUNT3, and HUNT4 in women and men with dementia compared to those without dementia at HUNT4. Data on adjustment variables are imputed using multiple imputation

	Model 1* MD (95% CI)	Model 2^†^ *MD* (95% CI)
Women		
HUNT1	0.87 (0.54, 1.20)	1.03 (0.70, 1.36)
HUNT2	0.47 (0.11, 0.83)	0.61 (0.26, 0.97)
HUNT3	− 0.18 (− 0.57, 0.21)	− 0.03 (− 0.42, 0.35)
HUNT4	− 0.90 (− 1.32, − 0.47)	− 0.86 (− 1.28, − 0.44)
Men		
HUNT1	0.67 (0.38, 0.95)	0.64 (0.36, 0.92)
HUNT2	0.56 (0.25, 0.87)	0.51 (0.20, 0.82)
HUNT3	0.18 (− 0.15, 0.53)	0.15 (− 0.19, 0.49)
HUNT4	− 0.31 (− 0.71, 0.09)	− 0.37 (− 0.77, 0.03)

*Model 1: Age-adjusted. ^†^Model 2: additional adjustment for educational attainment, marital status, physical activity, smoking, symptoms of anxiety or depression, and APOE ɛ4 status. Abbreviations: HUNT: The Trøndelag Health Study, HUNT1: 1984–86, HUNT2: 1995–97, HUNT3: 2006–08, HUNT4: 2017–19

### Waist circumference trajectories

The trajectories of WC from HUNT2 to HUNT4 among participants with and without dementia differed between women and men (Fig. 2, Table 4). At HUNT2, both women and men with dementia at HUNT4 had higher WC compared to those without dementia. At HUNT3, there was no difference in the WC of women with and without dementia, whereas men with dementia continued to have higher WC compared to men without dementia. At HUNT4, women with dementia had lower WC compared to women without dementia, whereas men with dementia continued to have higher WC than men without dementia, although the difference was negligible and not statistically significant (Fig. 2, Table 4).

**Table 4:** Absolute difference in mean waist circumference (WC) at HUNT2, HUNT3, and HUNT4, presented as mean difference (*MD*) with 95% CI in women and men with dementia compared to those without dementia at HUNT4

	Model 1* MD (95% CI)	Model 2^†^ *MD* (95% CI)
Women		
HUNT2	1.53 (0.62, 2.44)	1.97 (1.07, 2.87)
HUNT3	− 0.07 (− 1.10, 0.97)	0.35 (− 0.67, 1.36)
HUNT4	− 3.40 (− 4.88, − 1.92)	− 3.32 (− 4.77, − 1.86)
Men		
HUNT2	2.28 (1.41, 3.15)	2.06 (1.19, 2.94)
HUNT3	1.89 (0.95, 2.83)	1.73 (0.79, 2.67)
HUNT4	0.91 (− 0.62, 2.44)	0.68 (− 0.84, 2.20)

*Model 1: Age-adjusted. ^†^Model 2: additional adjustment for educational attainment, marital status, physical activity, smoking, symptoms of anxiety or depression, and APOE ɛ4 status. Abbreviations: HUNT: The Trøndelag Health Study, HUNT1: 1984–86, HUNT2: 1995–97, HUNT3: 2006–08, HUNT4: 2017–19

Data on adjustment variables are imputed using multiple imputation

### BMI and WC at the separate HUNT waves and dementia

**Fig. 3:**
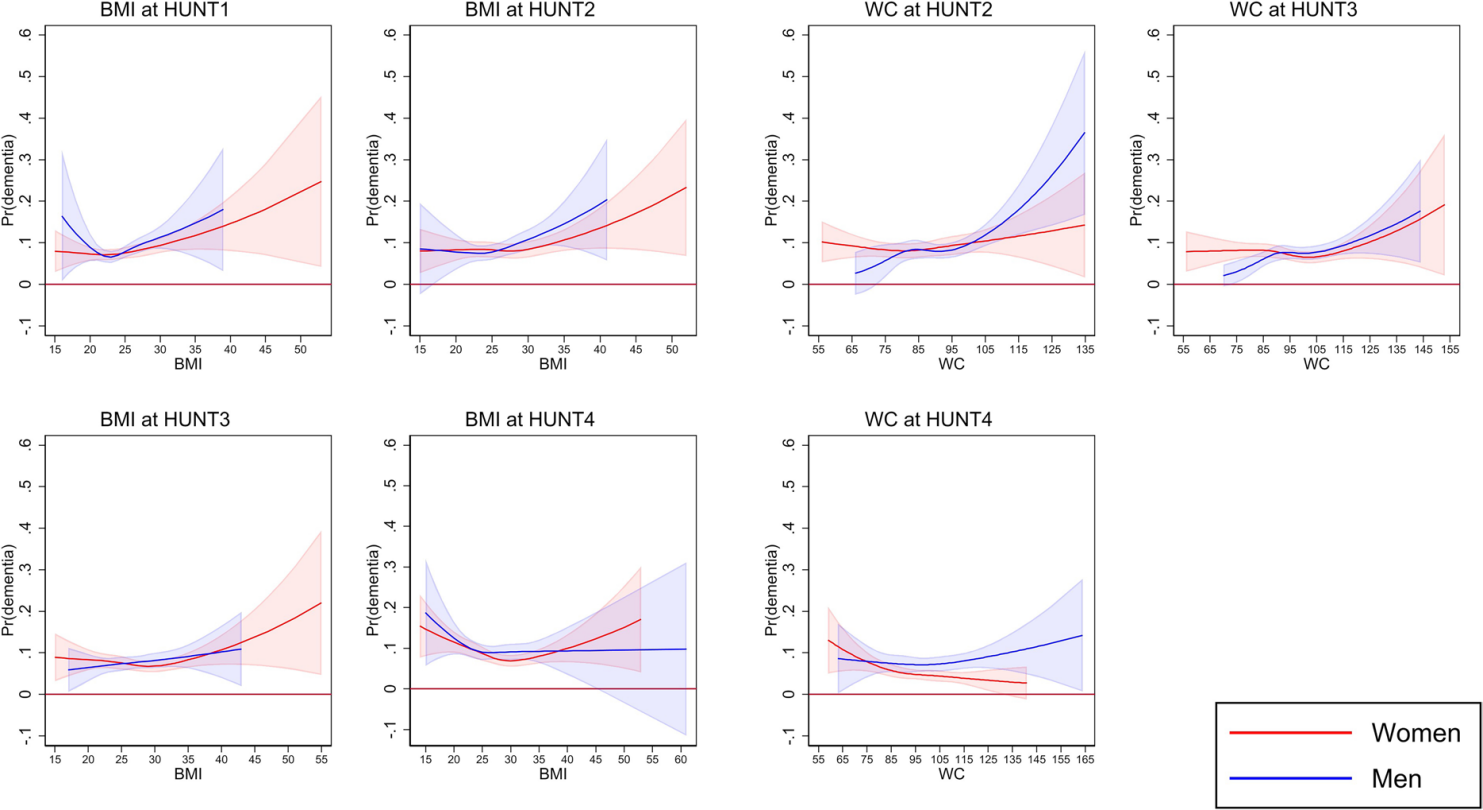
Predicted probability of dementia at HUNT4 by body mass index (BMI) and waist circumference (WC) for women and men at the four HUNT Study waves. Adjusted for age, educational attainment, marital status, physical activity, smoking, symptoms of anxiety or depression, and APOE ɛ4 status. Data on adjustment variables are imputed using multiple imputation. Abbreviations: HUNT: The Trøndelag Health Study, HUNT1: 1984–86, HUNT2: 1995–97, HUNT3: 2006–08, HUNT4: 2017–19

The logistic regression models with restricted cubic splines indicated non-linear relationships between BMI and WC at all four HUNT waves and dementia probability at HUNT4 for both sexes, apart from BMI at HUNT3 for men (Fig. 3). In men, BMI at HUNT1 had a U-shaped relationship with dementia probability, with the lowest probability around normal BMI. In women, a U-shaped relationship was seen first at HUNT4, with the lowest probabilities of dementia around BMI corresponding to obesity. For both sexes, the dementia probability associated with low BMI increased over time, whereas the probability related to high BMI decreased (Fig. 3). WC showed S-shaped curves for men at HUNT2 and both sexes at HUNT3, with increasing dementia probability at higher WC, particularly in men at HUNT2. At HUNT4, women had a cubic curve with decreasing dementia probability as WC increased, while men had a cubic curve with increasing dementia probability at WC above 100 cm (Fig. 3).

The association between categories of BMI and WC at HUNT1-4 and dementia at HUNT4 varied between women and men (Table S4). Higher dementia risk at HUNT4 was observed among men with overweight at HUNT1 (OR 1.29, 95% CI 1.05, 1.58), and in both women and men with obesity (women: OR 1.43, 95% CI 1.06, 1.94; men: OR 1.56, 95% CI 1.03, 2.36) at HUNT1. Men with obesity or high WC at HUNT2 had higher dementia risk (obesity: OR 1.74, 95% CI 1.26, 2.41; high WC: OR 1.97, 95% CI 1.48, 2.61), and there was also some evidence of higher dementia risk in men with overweight (OR 1.26, 95% CI 0.99, 1.61). There were no statistically significant associations between categories of BMI or WC at HUNT3 and dementia at HUNT4, although there was some evidence of higher dementia risk in men with high WC (OR 1.23, 95% CI 0.99, 1.54). Finally, at HUNT4, overweight was associated with lower dementia risk in both women (OR 0.74, 95% CI 0.60, 0.92) and men (OR 0.78, 95% CI 0.61, 0.98). High WC at HUNT4 was associated with lower dementia risk in women (OR 0.65, 95% CI 0.49, 0.85), but not in men. Underweight at HUNT1 (OR 8.95, 95% CI 1.92, 41.86) and HUNT2 (OR 7.27, 95% CI 1.38, 38.30) was associated with higher dementia risk in men, whereas in women underweight at HUNT4 was associated with higher dementia risk (OR 1.92, 95% CI 1.07, 3.46). However, due to the low number of underweight participants, particularly in the first two study waves, the estimates for underweight were severely underpowered (Table S4).

### Combined BMI and WC and dementia risk

**Fig. 4:**
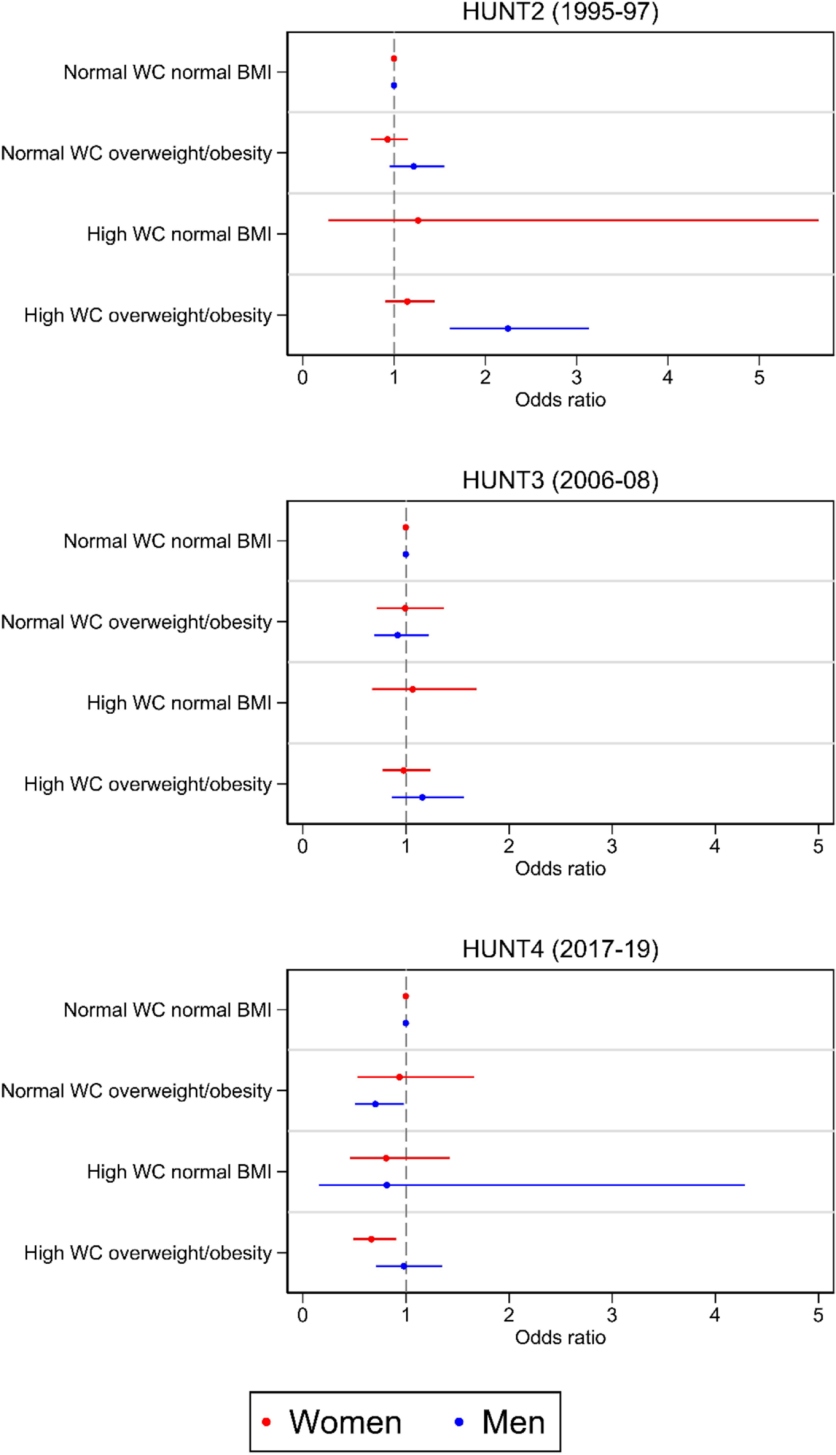
Odds ratios for dementia at HUNT4 associated with combinations of body mass index (BMI) and waist circumference (WC) at HUNT2, HUNT3, and HUNT4 for women and men. Normal WC normal BMI is the reference category. Adjusted for age, educational attainment, marital status, physical activity, smoking, symptoms of anxiety or depression, and APOE ɛ4 status. Data on adjustment variables are imputed using multiple imputation. Abbreviations: HUNT: The Trøndelag Health Study, HUNT2: 1995–97, HUNT3: 2006–08, HUNT4: 2017–19

Men with high WC combined with overweight or obesity at HUNT2 had higher dementia risk at HUNT4 (OR 2.25, 95% CI 1.61, 3.13), compared to men with normal WC and normal BMI (Fig. 4, Table S5). No combination of BMI and WC at HUNT3 was associated with dementia risk in women or men. At HUNT4, women with high WC combined with overweight or obesity had lower dementia risk (OR 0.67, 95% CI 0.49, 0.91) compared to women with normal WC and normal BMI, whereas men with normal WC combined with overweight or obesity had lower dementia risk (OR 0.71, 95% CI 0.51, 0.98). Data were insufficient to produce estimates for men with high WC and normal BMI at HUNT2 and HUNT3 (Fig. 4, Table S5).

### Sensitivity analyses

Results from the sensitivity analyses with complete cases are presented in Tables S6 through S9 in the supplementary materials. The results from the complete case analyses did not differ substantively from the main analyses with multiple imputation. The same applied to sensitivity analyses where participants with MCI (*N* = 3,412) were removed from the “no dementia” group (Tables S10 through S13).

## Discussion

In this study of 9,739 participants from the HUNT Study, BMI and WC trajectories differed by sex and dementia status. When assessed 22–33 years before dementia diagnosis (HUNT1-2), both men and women who later developed dementia had higher BMI and WC. Overweight, obesity, and high WC were associated with higher dementia risk, particularly in men. These associations shifted, and sex differences emerged, closer to dementia diagnosis. By 11 years before diagnosis (HUNT3), BMI differences diminished in both sexes, while WC remained higher in men, but not women, with dementia. At diagnosis (HUNT4), women with dementia had lower BMI and WC than those without, whereas differences in men were negligible. Overweight women and men were less likely to have dementia, whereas high WC was associated with a lower dementia risk in women, but not men, at time of diagnosis. Joint analyses of BMI and WC revealed distinct risk patterns: men had higher dementia risk when high WC and overweight/obesity were present decades before diagnosis, while late-life measures suggested lower risk in overweight or obesity combined with normal WC in men and high WC in women. These findings suggest that sex-specific factors may influence the relationship between adiposity measures over time and dementia risk, highlighting the importance of considering sex differences in dementia research.

Prior studies have suggested a reversal in the association of BMI with dementia across adulthood, with high midlife BMI increasing risk, while higher late-life BMI appears protective, likely due to reverse causation [7, 8, 15]. Our results reveal sex differences in the association of BMI with dementia risk, particularly later in life. Although both women and men with dementia had higher BMI 33 years before diagnosis, the shift toward a protective association occurred earlier in women. By 22 years before diagnosis, overweight and obesity were no longer significant risk factors for dementia in women, while obesity remained a risk factor in men, and a non-significant higher risk was also observed in men with overweight. BMI differences between men with and without dementia were smaller than in women both 11 years before diagnosis and at diagnosis, although overweight was associated with lower dementia risk in both sexes at diagnosis. Our results for underweight were underpowered but suggested that midlife underweight was a risk factor for dementia in men only, whereas late-life underweight was a risk factor for dementia in women only. Together, our findings suggest that high midlife BMI is a stronger predictor of dementia risk in men than in women, while preclinical dementia-associated weight loss may have a larger impact on women’s BMI trajectories.

Sex differences were more pronounced in the associations between WC and dementia. Women showed a reversal in the association between WC and dementia over time, similar to that seen for BMI, but this was not observed in men. Men with dementia had higher WC than those without 22–0 years before diagnosis, although differences at diagnosis were minimal. High WC at the time of diagnosis was linked to lower risk of dementia in women but not men. Joint analyses underscored the importance of WC as a dementia risk factor in men, where overweight and obesity 22 years before diagnosis was associated with higher dementia risk only when combined with high WC. Overall, our findings suggest that central adiposity may be an important risk factor for dementia in men. Further, the sex differences in the relation of WC with dementia at the time of diagnosis may indicate that dementia influences body composition and weight loss differently in women and men.

To our knowledge, this is the first study to examine sex differences in the longitudinal associations of mid-to-late-life BMI and WC with dementia. Our findings partially align with a German study showing lower dementia risk in women with overweight 10 years before diagnosis, with no such association in men [20]. However, in our study, the lower risk in overweight individuals was only evident when BMI was assessed at the time of dementia diagnosis. A Japanese study provided conflicting results, with higher dementia risk in women with overweight 8 years before diagnosis. The same study reported higher dementia risk in both women and men within the lowest WC quintiles [19]. Differences in body composition related to ethnicity may partly explain the discrepancies [28, 29], along with differences in mean age at baseline (59 years in the Japanese study [19] and 70 years in the German study[20]). Our findings help clarify sex differences in the association between anthropometric measures and dementia by including repeated measurements of BMI and WC, providing a longitudinal approach.

Biological factors, including hormonal differences and fat distribution, may underlie the observed sex differences. For instance, the time of menopause onset, which affects metabolism and increases the risk of adiposity [30, 31], has repeatedly been linked to dementia risk [32, 33]. Further, body fat distribution differs between women and men, with distribution being more peripheral in women, and more central in men [34, 35]. Central fat distribution, which is more commonly observed in men, is associated with several adverse metabolic effects, including insulin resistance, higher free fatty acids, and increased triglyceride levels. Peripheral fat distribution, on the other hand, is generally less associated with these metabolic disturbances [36]. These differences may shape how BMI and WC relate to dementia risk over time. Also, obesity increases the risk of diabetes, hypertension [26], and high LDL cholesterol [27], all of which are established midlife dementia risk factors [2]. Future research should consider mediation analyses to unravel the pathways linking adiposity to dementia in women and men.

This study benefits from a long follow-up period, a large study sample, clinical cognitive assessments of all study participants at ages 70+, and repeated objective anthropometric measurements over 35 years. Data on numerous confounders, including APOE ɛ4 status and a broad set of demographic, socioeconomic, and lifestyle-related confounders were available over the entire study period. However, the study also has several limitations. We did not have data on WC at HUNT1, limiting the ability of examining associations of early-midlife central adiposity with dementia risk. Furthermore, we did not have data on dementia or MCI at HUNT1-3 and participants had to be alive and present at HUNT4 70+ for assessment of cognitive status. As our study design does not include individuals who died before the age of 70 or were unable to participate for other reasons, our study sample likely consists of participants who are healthier than the general population. However, measures were taken (e.g. home and nursing home visits) to facilitate inclusion of individuals who were unable to attend the HUNT4 70+ examination site. Nevertheless, as obesity increases the risk of mortality [37], our results are likely influenced by survival bias. Also, despite the thoroughness of the clinical cognitive assessment, biomarker data was unavailable at the time of the assessment, increasing the risk of dementia misclassification. The homogenous HUNT population [21] limits generalizability to other populations. Finally, since this is an observational study, there may be unmeasured confounders. This poses a risk that the observed associations are affected by residual confounding, meaning they might not indicate a causal relationship.

## Conclusion

Our study demonstrates that BMI and WC trajectories over adulthood are significantly associated with dementia risk at age 70 and older, with notable sex-specific differences, particularly for late-life BMI and WC. Moreover, the joint effects of BMI and WC provide additional insights into the complex interplay between body composition and dementia risk. These findings underscore the importance of lifelong body composition monitoring and tailored interventions to reduce dementia risk, highlighting the need for further research to better understand the underlying mechanisms and to develop sex-specific prevention strategies.

## Supplementary Information

Below is the link to the electronic supplementary material.

Supplementary file1 (DOCX 137 KB)

## Data Availability

The Trøndelag Health Study (HUNT) has invited persons aged 13 - 100 years to four surveys between 1984 and 2019. Comprehensive data from more than 140,000 persons having participated at least once and biological material from 78,000 persons are collected. The data are stored in HUNT databank and biological material in HUNT biobank. HUNT Research Centre has permission from the Norwegian Data Inspectorate to store and handle these data. The key identication in the data base is the personal identication number given to all Norwegians at birth or immigration, whilst de-identied data are sent to researchers upon approval of a research protocol by the Regional Ethical Committee and HUNT Research Centre. To protect participants’ privacy, HUNT Research Centre aims to limit storage of data outside HUNT databank, and cannot deposit data in open repositories. HUNT databank has precise information on all data exported to dierent projects and are able to reproduce these on request. There are no restrictions regarding data export given approval of applications to HUNT Research Centre. For more information see: http://www.ntnu.edu/hunt/data
