# Correction to: Sex differences in body mass index and waist circumference trajectories and dementia risk: the HUNT4 70+ study

**DOI:** 10.1007/s11357-025-01753-z

**Published:** 2025-06-17

**Authors:** Ekaterina Zotcheva, Bjørn Heine Strand, Vegard Skirbekk, Kay Deckers, Steinar Krokstad, Gill Livingston, Archana Singh‑Manoux, Geir Selbæk

**Affiliations:** 1 Norwegian National Centre for Ageing and Health, Vestfold Hospital Trust, Aldring Og Helse, PO Box 2136, 3103 Tonsberg, Norway; 2 Department of Geriatric Medicine, Oslo University Hospital, Nydalen, OUS HF, Ulleval Sykehus, PO Box 4956, 0424 Oslo, Norway; 3 Department of Physical Health and Ageing, Norwegian Institute of Public Health, Skoyen, PO Box 222, 0213 Oslo, Norway; 4 Centre for Fertility and Health, Norwegian Institute of Public Health, PO Box 222, 0213 Skoyen Oslo, Norway; 5 Department of Psychiatry and Neuropsychology, Mental Health and Neuroscience Research Institute (MHeNs), Alzheimer Centrum Limburg, Maastricht University, PO Box 616, 6200 MD Maastricht, The Netherlands; 6 Department of Public Health and Nursing, Faculty of Medicine and Health Sciences, HUNT Research Centre, Norwegian University of Science and Technology, PO Box 8905, 7491 Trondheim, Norway; 7 Levanger Hospital, Nord-Trondelag Hospital Trust, PO Box 333, 7601 Levanger, Norway; 8 Division of Psychiatry, University College London, 149 Tottenham Ct Rd, London, W1 T7 NF UK; 9 North London NHS Foundation Trust, 4 St Pancras Way, London, NW1 OPE UK; 10 Epidemiology of Ageing and Neurodegenerative Diseases, U1153 Inserm, Universite Paris Cite, 10 Avenue de Villemin, 75010 Paris, France; 11 Institute of Clinical Medicine, Faculty of Medicine, University of Oslo, Blindern, PO Box 1072, 0316 Oslo, Norway

**Correction to: GeroScience**

10.1007/s11357-025-01660-3

The original version of this article unfortunately contained an error in the description of waist circumference (WC) measurement during the fourth wave of the Trøndelag Health Study (HUNT4).

In page 3, under Body mass index and waist circumference section, "WC was measured at the height of the umbilicus to the nearest whole cm with the person in a standing position with arms hanging relaxed. WC was measured three times; at HUNT2, 3, and 4 (...)". However, for most participants at HUNT4, WC was measured using the InBody 770 bioimpedance body composition analyzer. The correct statement should therefore be: "WC was measured at the height of the umbilicus to the nearest whole cm with the person in a standing position with arms hanging relaxed at HUNT2 and HUNT3, and using the InBody 770 bioimpedance body composition analyzer at HUNT4. Participants who were unable to stand or had pacemakers were measured manually at HUNT4, using the same procedure as at HUNT2 and HUNT3."

