# Supplementary material for: Sex differences in body mass index and waist circumference trajectories and dementia risk: the HUNT4 70+ study
Source: GeroScience. 2025 Apr 22;48(1):405–19. doi: 10.1007/s11357-025-01660-3 (PMC12972498; doi:10.1007/s11357-025-01660-3)
Supplement: Supplementary file 1 — Supplementary file1 (DOCX 137 KB) [file 11357_2025_1660_MOESM1_ESM.docx]

**Supplementary materials for Sex differences in body mass index and waist circumference trajectories and dementia risk: The HUNT4 70+ Study**

*GeroScience*

Ekaterina Zotcheva*, Bjørn Heine Strand, Vegard Skirbekk, Kay Deckers, Steinar Krokstad, Gill Livingston, Archana Singh-Manoux, and Geir Selbæk

*Corresponding author. E-mail address: [ekaterina.zotcheva@aldringoghelse.no](mailto:ekaterina.zotcheva@aldringoghelse.no). Postal address: Aldring og helse, PO Box 2136, 3103 Tønsberg, Norway

**Contents**

[**Fig S1** 2](#_Toc190951779)

[**Other covariates** 3](#_Toc190951780)

[*Time invariant covariates* 3](#_Toc190951781)

[*Time-dependent covariates* 3](#_Toc190951782)

[**Table S1** 4](#_Toc190951783)

[**Table S2** 5](#_Toc190951784)

[**Table S3** 5](#_Toc190951785)

[**Table S4** 6](#_Toc190951786)

[**Table S5** 6](#_Toc190951787)

[**Complete case analyses** 7](#_Toc190951788)

[**Table S6** 7](#_Toc190951789)

[**Table S7** 7](#_Toc190951790)

[**Table S8** 8](#_Toc190951791)

[**Table S9** 8](#_Toc190951792)

[**Analyses with MCI participants removed** 9](#_Toc190951793)

[**Table S10** 9](#_Toc190951794)

[**Table S11** 9](#_Toc190951795)

[**Table S12** 10](#_Toc190951796)

[**Table S13** 10](#_Toc190951797)

[**References** 11](#_Toc190951798)


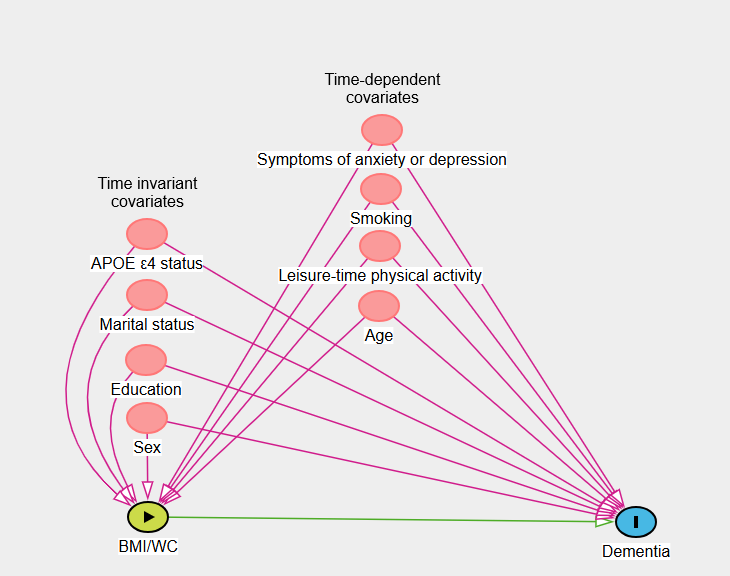


**Fig S1** Directed acyclic graph (DAG) illustrating the association of BMI and WC at HUNT1-4 (green circle) with dementia at HUNT4 (blue circle) and potential confounding from time invariant covariates and time-dependent covariates (pink circles). Pink lines indicate biasing paths, whereas green lines indicate causal paths. DAG created using [www.dagitty.net](http://www.dagitty.net)

# **Other covariates**

## *Time invariant covariates*

Education was self-reported via questionnaires at HUNT1, HUNT2, and HUNT4. Education was grouped into primary , secondary, or tertiary. We used the earliest availiable data on education for each participant in the analyses. Marital status was self-reported at HUNT1. Participants reported whether they were unmarried, married, widow(er), separated, or divorced. Information on martial status was grouped into three categories (unmarried or divorced/married/widow[er]) for the statistical analyses. DNA was extracted from whole blood for genotyping at HUNT2, HUNT3, and HUNT4. All genotyping was performed at the Genomics-Core Facility at the Norwegian University of Science and Technology, NTNU. APOE ε4 carrier status was assessed using the two single-nucleotide polymorphisms (SNPs), rs7412 and rs429358. Participants were thereafter categorized according to their APOE ε4 carrier status into three groups (no ε4 allele/ε4 heterozygote/ε4 homozygote).

## *Time-dependent covariates*

Physical activity was self-reported by participants at HUNT1-4. At HUNT1, HUNT2, and HUNT4, participants answered three questions regarding the frequency, duration, and intensity of their leisure-time physical activity during a typical week in the last year. At HUNT2, participants self-reported the average number of hours in low and vigorous intensity physical activity per week in the last year. Based on these questions, participants were categorized according to whether they were physically active at any intensity for at least 1 hour per week (less than 1 hour weekly physical activity/1 hour or more weekly physical activity).

Smoking was self-reported by participants at HUNT1-4 as either current daily smoker, former daily smoker, or never daily smoker. A dichotomous measure of daily smoking was used in the statistical analyses (never or former daily smoker/current daily smoker).

Symptoms of anxiety and depression were assessed using the Anxiety and Depression Index 4 (ADI-4) [1] at HUNT1 and the Hospital Anxiety and Depression Scale [2] at HUNT2-4. Participants with an ADI-4 score at or above the 88^th^ percentile [1] at HUNT1, and with a HADS depression or anxiety subscale score at or above 8 [2] at HUNT2-4 were categorized as participants with prevalent symptoms of anxiety or depression at the respective HUNT survey, whereas those scoring below these cut-offs were categorized as not having symptoms of anxiety or depression.

| **Table S1**. Missing values on potential confounders for the study population (n=9,739). | |
| --- | --- |
| Variable | Missing, n (%) |
| Marital status | 1,029 (10.6) |
| APOE ɛ4 presence | 95 (1.0) |
| Level of physical activity |  |
| HUNT1 | 2,313 (23.8) |
| HUNT2 | 1,745 (17.9) |
| HUNT3 | 1,595 (16.4) |
| HUNT4 | 1,049 (10.8) |
| Smoking status |  |
| HUNT1 | 1,610 (16.5) |
| HUNT2 | 1,212 (12.4) |
| HUNT3 | 682 (7.0) |
| HUNT4 | 2,204 (22.6) |
| Symptoms of anxiety or depression |  |
| HUNT1 | 2,463 (25.3) |
| HUNT2 | 2,445 (25.1) |
| HUNT3 | 2,517 (25.8) |
| HUNT4 | 2,203 (22.6) |
| Abbreviations: APOE: apolipoprotein E, HUNT: The Trøndelag Health Study, HUNT1: 1984-86, HUNT2: 1995-97, HUNT3: 2006-08, HUNT4: 2017-19. | |

| **Table S2**. Time-dependent characteristics at the four HUNT surveys for participants with BMI and/or WC data at each survey, stratified by dementia status at HUNT4. | | | | | | | | |
| --- | --- | --- | --- | --- | --- | --- | --- | --- |
|  | No dementia at HUNT4 | | | | Dementia at HUNT4 | | | |
|  | HUNT1 (1984-86) | HUNT2  (1995-97) | HUNT3  (2006-08) | HUNT4  (2017-19) | HUNT1 (1984-86) | HUNT2  (1995-97) | HUNT3  (2006-08) | HUNT4  (2017-19) |
| N with BMI and/or WC data (%) | 7,304 (88.7) | 7,273 (88.4) | 7,244 (88.0) | 8,133 (98.8) | 1,360 (90.3) | 1,317 (87.4) | 1,143 (75.9) | 1,186 (78.7) |
| Age, mean (SD) | 44.2 (5.7) | 55.3 (5.7) | 66.2 (5.6) | 77.0 (5.6) | 51.0 (7.5) | 62.1 (7.5) | 72.4 (7.1) | 82.9 (7.2) |
| <1 hr weekly physical activity, n (%) | 1,785 (24.4) | 5,431 (75.7) | 4,173 (57.6) | 4,580 (56.3) | 336 (24.7) | 830 (63.0) | 569 (49.8) | 313 (26.4) |
| Daily smoker, n (%) | 1,594 (21.8) | 1,501 (20.6) | 812 (11.2) | 489 (6.0) | 312 (22.9) | 268 (20.4) | 121 (10.6) | 64 (5.4) |
| Symptoms of anxiety or depression, n (%) | 723 (9.9) | 1,253 (17.2) | 1,045 (14.4) | 1,175 (14.5) | 174 (12.8) | 256 (19.4) | 231 (20.2) | 242 (20.4) |
| BMI, mean (SD) | 24.7 (3.2) | 26.7 (3.6) | 27.7 (4.1) | 27.3 (4.4) | 25.5 (3.7) | 27.3 (4.1) | 27.7 (4.3) | 26.6 (4.8) |
| Underweight, n (%) | 40 (0.6) | 17 (0.2) | 22 (0.3) | 67 (0.8) | 8 (0.6) | 9 (0.7) | 8 (0.7) | 28 (2.4) |
| Normal BMI, n (%) | 4,294 (58.8) | 2,394 (32.9) | 1,770 (24.4) | 2,419 (29.7) | 640 (47.1) | 363 (27.6) | 289 (25.3) | 432 (36.4) |
| Overweight, n (%) | 2,532 (34.7) | 3,720 (51.2) | 3,675 (50.7) | 3,754 (46.2) | 579 (42.6) | 669 (50.8) | 535 (46.8) | 475 (40.1) |
| Obesity, n (%) | 438 (6.0) | 1,140 (15.7) | 1,770 (24.4) | 1,889 (23.2) | 133 (9.8) | 275 (20.9) | 305 (26.7) | 248 (20.9) |
| WC, mean (SD) |  | 86.8 (10.6) | 95.4 (11.4) | 97.2 (13.1) |  | 88.2 (11.2) | 95.9 (11.7) | 95.8 (14.3) |
| High WC, n (%) |  | 1,276 (17.5) | 3,439 (47.5) | 4,096 (50.4) |  | 354 (26.9) | 597 (52.2) | 270 (22.8) |
| Abbreviations: APOE: apolipoprotein E, BMI: body mass index (kg/m^2^), HUNT: The Trøndelag Health Study, SD: standard deviations, WC: waist circumference. | | | | | | | | |

| **Table S3**. Time invariant characteristics of the study sample (n=9,739) stratified by dementia status at HUNT4. | | |
| --- | --- | --- |
|  | No dementia at HUNT4 | Dementia at HUNT4 |
| N (%) | 8,232 (84.5) | 1,507 (15.5) |
| Women, n (%) | 4,409 (53.6) | 890 (59.1) |
| Education*, n (%) |  |  |
| Primary | 3,904 (47.4) | 996 (66.1) |
| Secondary | 2,555 (31.0) | 385 (25.6) |
| Tertiary | 1,773 (18.5) | 126 (8.4) |
| Married^†^, n (%) | 6,615 (80.4) | 1,197 (79.4) |
| APOE ε4 presence, n (%) | 2,347 (28.5) | 563 (37.4) |
| *At HUNT1, HUNT2, and/or HUNT4. ^†^At HUNT1. Abbreviations: APOE: apolipoprotein E, HUNT: The Trøndelag Health Study. | | |

| **Table S4**. Associations of categorical BMI at HUNT1-4 and WC at HUNT2-4 with dementia diagnosis at HUNT4 in women and men. Performed on multiple imputed data. | | | | |
| --- | --- | --- | --- | --- |
|  | Women | | Men | |
|  | Cases/*n* | OR (95% CI)* | Cases/*n* | OR (95% CI)* |
| **HUNT1** |  |  |  |  |
| Underweight | 5/42 | 0.65 (0.22, 1.94) | 3/6 | 8.95 (1.92, 41.84) |
| Normal | 407/3,002 | 1.00 (ref.) | 233/1,932 | 1.00 (ref.) |
| Overweight | 304/1,389 | 1.19 (0.98, 1.45) | 275/1,722 | 1.29 (1.05, 1.58) |
| Obesity | 96/372 | 1.43 (1.06, 1.94) | 37/199 | 1.56 (1.03, 2.36) |
| **HUNT2** |  |  |  |  |
| Underweight | 7/20 | 1.66 (0.54, 5.07) | 2/6 | 7.27 (1.38, 38.30) |
| Normal | 237/1,705 | 1.00 (ref.) | 126/1,052 | 1.00 (ref.) |
| Overweight | 361/2,143 | 0.97 (0.79, 1.19) | 308/2,246 | 1.26 (0.99, 1.61) |
| Obesity | 186/903 | 1.10 (0.86, 1.42) | 89/512 | 1.74 (1.26, 2.40) |
| Normal WC | 530/3,544 | 1.00 (ref.) | 433/3,408 | 1.00 (ref.) |
| High WC | 262/1,224 | 1.20 (0.98, 1.46) | 92/406 | 1.97 (1.48, 2.61) |
| **HUNT3** |  |  |  |  |
| Underweight | 8/25 | 1.13 (0.33, 3.92) | 0/5 | N/A |
| Normal | 182/1,296 | 1.00 (ref.) | 107/763 | 1.00 (ref.) |
| Overweight | 279/2,021 | 0.91 (0.72, 1.14) | 256/2,189 | 0.96 (0.74, 1.26) |
| Obesity | 200/1,250 | 1.06 (0.82, 1.37) | 105/825 | 1.09 (0.79, 1.51) |
| Normal WC | 240/1,763 | 1.00 (ref.) | 301/2,579 | 1.00 (ref.) |
| High WC | 431/2,829 | 0.98 (0.81, 1.20) | 166/1,207 | 1.23 (0.99, 1.54) |
| **HUNT4** |  |  |  |  |
| Underweight | 24/80 | 1.92 (1.07, 3.46) | 4/15 | 1.98 (0.54, 7.33) |
| Normal | 268/1,685 | 1.00 (ref.) | 164/1,166 | 1.00 (ref.) |
| Overweight | 241/2,029 | 0.74 (0.60, 0.92) | 234/2,200 | 0.78 (0.61, 0.98) |
| Obesity | 153/1,238 | 0.79 (0.62, 1.02) | 95/899 | 0.83 (0.61, 1.14) |
| Normal WC | 120/1,396 | 1.00 (ref.) | 173/2,277 | 1.00 (ref.) |
| High WC | 143/2,801 | 0.65 (0.49, 0.85) | 127/1,565 | 1.16 (0.89, 1.52) |
| *Adjusted for age, educational attainment, marital status, physical activity, smoking, symptoms of anxiety or depression, and APOE ɛ4 status. Abbreviations: BMI: body mass index, CI: confidence intervals, HUNT: The Trøndelag Health Study, HUNT1: 1984-86, HUNT2: 1995-97, HUNT3: 2006-08, HUNT4: 2017-19, OR: odds ratio, WC: waist circumference. | | | | |

| **Table S5**. Associations of different combinations of BMI and WC and HUNT2, HUNT3, and HUNT4 with dementia risk at HUNT4 in women and men. Performed on multiple imputed data. | | | | |
| --- | --- | --- | --- | --- |
|  | Women | | Men | |
|  | Cases/n | OR (95% CI)* | Cases/n | OR (95% CI)* |
| **HUNT2** |  |  |  |  |
| Normal WC, normal BMI | 233/1,691 | 1.00 (ref.) | 125/1,051 | 1.00 (ref.) |
| Normal WC, overweight/obesity | 290/1,832 | 0.93 (0.75, 1.15) | 306/2,350 | 1.22 (0.95, 1.55) |
| High WC, normal BMI | 4/13 | 1.26 (0.28, 5.65) | 1/1 | N/A |
| High WC, overweight/obesity | 257/1,210 | 1.15 (0.91, 1.44) | 91/405 | 2.25 (1.61, 3.13) |
| **HUNT3** |  |  |  |  |
| Normal WC, normal BMI | 138/1,083 | 1.00 (ref.) | 104/760 | 1.00 (ref.) |
| Normal WC, overweight/obesity | 92/652 | 0.99 (0.72, 1.37) | 195/1,809 | 0.92 (0.70, 1.22) |
| High WC, normal BMI | 43/212 | 1.07 (0.68, 1.69) | 2/2 | N/A |
| High WC, overweight/obesity | 386/2,615 | 0.98 (0.77, 1.24) | 164/1,202 | 1.16 (0.86, 1.56) |
| **HUNT4** |  |  |  |  |
| Normal WC, normal BMI | 87/1,123 | 1.00 (ref.) | 87/994 | 1.00 (ref.) |
| Normal WC, overweight/obesity | 19/211 | 0.94 (0.53, 1.66) | 83/1,273 | 0.71 (0.51, 0.98) |
| High WC, normal BMI | 16/268 | 0.81 (0.46, 1.43) | 2/17 | 0.82 (0.16, 4.29) |
| High WC, overweight/obesity | 126/2,531 | 0.67 (0.49, 0.91) | 125/1,546 | 0.98 (0.71, 1.35) |
| *Adjusted for age, educational attainment, marital status, physical activity, smoking, symptoms of anxiety or depression, and APOE ɛ4 status. Abbreviations: BMI: body mass index, CI: confidence intervals, HUNT: The Trøndelag Health Study, HUNT2: 1995-97, HUNT3: 2006-08, HUNT4: 2017-19, OR: odds ratio, WC: waist circumference. | | | | |

# **Complete case analyses**

| **Table S6**. Absolute difference in mean BMI at HUNT1, HUNT2, HUNT3, and HUNT4 with 95% CI in women and men with dementia compared to those without dementia at HUNT4. Performed on complete case data for BMI analyses (n=8,450). | | |
| --- | --- | --- |
|  | Model 1*  *MD* (95% CI) | Model 2^†^  *MD* (95% CI) |
| **Women (n=4,654)** |  |  |
| HUNT1 | 0.89 (0.53, 1.25) | 1.05 (0.69, 1.41) |
| HUNT2 | 0.47 (0.07, 0.88) | 0.61 (0.21, 1.01) |
| HUNT3 | -0.18 (-0.62, 0.26) | -0.05 (-0.49, 0.38) |
| HUNT4 | -0.83 (-1.40, -0.27) | -0.87 (-1.43, -0.31) |
| **Men (n=3,796)** |  |  |
| HUNT1 | 0.73 (0.42, 1.04) | 0.70 (0.39, 1.01) |
| HUNT2 | 0.59 (0.24, 0.94) | 0.54 (0.19, 0.89) |
| HUNT3 | 0.12 (-0.26, 0.50) | 0.09 (-0.29, 0.47) |
| HUNT4 | -0.07 (-0.60, 0.47) | -0.07 (-0.60, 0.47) |
| *Model 1: Age-adjusted. ^†^Model 2: additional adjustment for educational attainment, marital status, physical activity, smoking, symptoms of anxiety or depression, and APOE ɛ4 status. Abbreviations: HUNT: The Trøndelag Health Study, HUNT1: 1984-86, HUNT2: 1995-97, HUNT3: 2006-08, HUNT4: 2017-19. | | |

| **Table S7**. Absolute difference in mean waist circumference at HUNT2, HUNT3, and HUNT4 with 95% CI in women and men with dementia compared to those without dementia at HUNT4. Performed on complete case data for WC analyses (n=8,051). | | |
| --- | --- | --- |
|  | Model 1*  *MD* (95% CI) | Model 2^†^  *MD* (95% CI) |
| **Women (n=4,378)** |  |  |
| HUNT2 | 1.46 (0.38, 2.54) | 1.86 (0.79, 2.92) |
| HUNT3 | -0.15 (-1.35, 1.05) | 0.18 (-0.99, 1.34) |
| HUNT4 | -3.59 (-5.41, -1.76) | -3.83 (-5.62, -2.04) |
| **Men (n=3,673)** |  |  |
| HUNT2 | 2.14 (1.17, 3.11) | 1.89 (0.92, 2.86) |
| HUNT3 | 1.68 (0.61, 2.75) | 1.48 (0.42, 2.54) |
| HUNT4 | 0.86 (-0.96, 2.69) | 0.76 (-1.05, 2.57) |
| *Model 1: Age-adjusted. ^†^Model 2: additional adjustment for educational attainment, marital status, physical activity, smoking, symptoms of anxiety or depression, and APOE ɛ4 status. Abbreviations: HUNT: The Trøndelag Health Study, HUNT2: 1995-97, HUNT3: 2006-08, HUNT4: 2017-19. | | |

| **Table S8**. Associations of categorical BMI at HUNT1-4 and WC at HUNT2-4 with dementia diagnosis at HUNT4 in women and men. Performed on complete case data. | | | | |
| --- | --- | --- | --- | --- |
|  | Women | | Men | |
|  | Cases/n | OR (95% CI)* | Cases/n | OR (95% CI)* |
| **HUNT1** |  |  |  |  |
| Underweight | 3/34 | 0.51 (0.14, 1.90) | 3/5 | 14.46 (2.55, 82.02) |
| Normal | 311/2,426 | 1.00 (ref.) | 187/1,613 | 1.00 (ref.) |
| Overweight | 238/1,111 | 1.21 (0.97, 1.50) | 219/1,404 | 1.29 (1.02, 1.51) |
| Obesity | 72/293 | 1.50 (1.07, 2.10) | 30/162 | 1.70 (1.08, 2.67) |
| **HUNT2** |  |  |  |  |
| Underweight | 4/12 | 1.62 (0.41, 6.44) | 1/4 | 6.95 (0.63, 76.27) |
| Normal | 139/1,257 | 1.00 (ref.) | 99/860 | 1.00 (ref.) |
| Overweight | 200/1,534 | 0.96 (0.74, 1.24) | 224/1,803 | 1.20 (0.91, 1.59) |
| Obesity | 118/647 | 1.19 (0.88, 1.61) | 59/405 | 1.45 (0.99, 2.13) |
| Normal WC | 310/2,589 | 1.00 (ref.) | 318/2,753 | 1.00 (ref.) |
| High WC | 151/858 | 1.16 (0.91, 1.48) | 65/317 | 1.94 (1.40, 2.70) |
| **HUNT3** |  |  |  |  |
| Underweight | 5/18 | 1.34 (0.34, 5.35) | 0/3 | N/A |
| Normal | 118/983 | 1.00 (ref.) | 78/582 | 1.00 (ref.) |
| Overweight | 181/1,545 | 0.93 (0.70, 1.23) | 178/1,663 | 0.94 (0.68, 1.30) |
| Obesity | 124/927 | 1.04 (0.76, 1.42) | 69/605 | 1.03 (0.70, 1.53) |
| Normal WC | 158/1,361 | 1.00 (ref.) | 212/1,967 | 1.00 (ref.) |
| High WC | 272/2,113 | 0.97 (0.76, 1.23) | 111/885 | 1.24 (0.95, 1.62) |
| **HUNT4** |  |  |  |  |
| Underweight | 10/49 | 2.07 (0.93, 4.58) | 0/4 | N/A |
| Normal | 106/1,085 | 1.00 (ref.) | 73/774 | 1.00 (ref.) |
| Overweight | 84/1,334 | 0.64 (0.46, 0.89) | 108/1,490 | 0.74 (0.53, 1.03) |
| Obesity | 62/821 | 0.74 (0.52, 1.07) | 47/624 | 0.82 (0.54, 1.26) |
| Normal WC | 68/979 | 1.00 (ref.) | 103/1,600 | 1.00 (ref.) |
| High WC | 76/2,004 | 0.58 (0.41, 0.83) | 87/1,130 | 1.31 (0.95, 1.82) |
| *Adjusted for age, educational attainment, marital status, level of physical activity, smoking, symptoms of anxiety or depression, and APOE ɛ4 status. Abbreviations: BMI: body mass index, CI: confidence intervals, HUNT: The Trøndelag Health Study, HUNT1: 1984-86, HUNT2: 1995-97, HUNT3: 2006-08, HUNT4: 2017-19, OR: odds ratio, WC: waist circumference. | | | | |

| **Table S9**. Associations of different combinations of BMI and WC and HUNT2, HUNT3, and HUNT4 with dementia risk at HUNT4 in women and men. Performed on complete case data. | | | | |
| --- | --- | --- | --- | --- |
|  | Women | | Men | |
|  | Cases/n | OR (95% CI)* | Cases/n | OR (95% CI)* |
| **HUNT2** |  |  |  |  |
| Normal WC, normal BMI | 136/1,248 | 1.00 (ref.) | 98/859 | 1.00 (ref.) |
| Normal WC, overweight/obesity | 170/1,328 | 0.97 (0.75, 1.27) | 219/1,889 | 1.13 (0.86, 1.49) |
| High WC, normal BMI | 3/8 | 1.82 (0.45, 7.42) | 1/1 | N/A |
| High WC, overweight/obesity | 148/850 | 1.14 (0.86, 1.53) | 64/316 | 2.09 (1.42, 3.08) |
| **HUNT3** |  |  |  |  |
| Normal WC, normal BMI | 91/830 | 1.00 (ref.) | 77/581 | 1.00 (ref.) |
| Normal WC, overweight/obesity | 61/511 | 0.98 (0.66, 1.46) | 134/1,381 | 0.87 (0.63, 1.22) |
| High WC, normal BMI | 26/152 | 0.96 (0.54, 1.69) | 0/0 | N/A |
| High WC, overweight/obesity | 244/1,959 | 0.96 (0.73, 1.28) | 111/884 | 1.13 (0.80, 1.61) |
| **HUNT4** |  |  |  |  |
| Normal WC, normal BMI | 50/795 | 1.00 (ref.) | 54/704 | 1.00 (ref.) |
| Normal WC, overweight/obesity | 10/140 | 0.92 (0.42, 2.00) | 49/893 | 0.65 (0.43, 0.98) |
| High WC, normal BMI | 10/193 | 0.88 (0.43, 1.82) | 2/16 | 1.12 (0.22, 5.76) |
| High WC, overweight/obesity | 66/1,811 | 0.59 (0.39, 0.87) | 85/1,113 | 1.04 (0.70, 1.53) |
| *Adjusted for age, educational attainment, marital status, level of physical activity, smoking, symptoms of anxiety or depression, and APOE ɛ4 status. Abbreviations: BMI: body mass index, CI: confidence intervals, HUNT: The Trøndelag Health Study, HUNT2: 1995-97, HUNT3: 2006-08, HUNT4: 2017-19, OR: odds ratio, WC: waist circumference. | | | | |

# **Analyses with MCI participants removed**

| **Table S10**. Absolute difference in mean BMI at HUNT1, HUNT2, HUNT3, and HUNT4 with 95% CI in women and men with dementia compared to those without cognitive impairment at HUNT4. Performed on multiple imputed data. | | |
| --- | --- | --- |
|  | Model 1* *MD* (95% CI) | Model 2^†^ *MD* (95% CI) |
| **Women (n=3,556)** |  |  |
| HUNT1 | 0.98 (0.62, 1.35) | 1.18 (0.81, 1.54) |
| HUNT2 | 0.55 (0.15, 0.94) | 0.71 (0.31, 1.10) |
| HUNT3 | -0.18 (-0.60, 0.25) | -0.02 (-0.45, 0.40) |
| HUNT4 | -1.03 (-1.49, -0.57) | -1.03 (-1.49, -0.57) |
| **Men (n=2,771)** |  |  |
| HUNT1 | 0.75 (0.44, 1.06) | 0.76 (0.46, 1.07) |
| HUNT2 | 0.63 (0.27, 0.97) | 0.61 (0.27, 0.94) |
| HUNT3 | 0.25 (-0.11, 0.62) | 0.24 (-0.13, 0.60) |
| HUNT4 | -0.27 (-0.70, 0.16) | -0.32 (-0.74, 0.10) |
| *Model 1: Age-adjusted. ^†^Model 2: additional adjustment for educational attainment, marital status, physical activity, smoking, symptoms of anxiety or depression, and APOE ɛ4 status. Abbreviations: HUNT: The Trøndelag Health Study, HUNT1: 1984-86, HUNT2: 1995-97, HUNT3: 2006-08, HUNT4: 2017-19. | | |

| **Table S11**. Absolute difference in mean waist circumference at HUNT2, HUNT3, and HUNT4 with 95% CI in women and men with dementia compared to those without cognitive impairment at HUNT4. Performed on multiple imputed data. | | |
| --- | --- | --- |
|  | Model 1* *MD* (95% CI) | Model 2^†^ *MD* (95% CI) |
| **Women (n=3,497)** |  |  |
| HUNT2 | 2.07 (1.08, 3.07) | 2.52 (1.52, 3.52) |
| HUNT3 | 0.33 (-0.79, 1.44) | 0.75 (-0.36, 1.86) |
| HUNT4 | -3.46 (-4.98, -1.93) | -3.42 (-4.93, -1.91) |
| **Men (n=2,729)** |  |  |
| HUNT2 | 2.61 (1.67, 3.54) | 2.43 (1.49, 3.38) |
| HUNT3 | 2.12 (1.11, 3.14) | 2.00 (0.97, 3.02) |
| HUNT4 | 1.30 (-0.29, 2.90) | 1.10 (-0.49, 2.69) |
| *Model 1: Age-adjusted. ^†^Model 2: additional adjustment for educational attainment, marital status, physical activity, smoking, symptoms of anxiety or depression, and APOE ɛ4 status. Abbreviations: HUNT: The Trøndelag Health Study, HUNT2: 1995-97, HUNT3: 2006-08, HUNT4: 2017-19. | | |

| **Table S12**. Associations of categorical BMI at HUNT1-4 and WC at HUNT2-4 with dementia diagnosis vs. no cognitive impairment at HUNT4 in women and men. Performed on multiple imputed data. | | | | |
| --- | --- | --- | --- | --- |
|  | Women | | Men | |
|  | Cases/*n* | OR (95% CI)* | Cases/*n* | OR (95% CI)* |
| **HUNT1** |  |  |  |  |
| Underweight | 5/25 | 1.14 (0.38, 3.42) | 3/5 | 7.85 (1.43, 43.17) |
| Normal | 407/2,040 | 1.00 (ref.) | 233/1,215 | 1.00 (ref.) |
| Overweight | 304/922 | 1.23 (0.99, 1.54) | 275/1,066 | 1.33 (1.06, 1.67) |
| Obesity | 96/253 | 1.53 (1.10, 2.14) | 37/107 | 2.01 (1.24, 3.24) |
| **HUNT2** |  |  |  |  |
| Underweight | 7/12 | 2.88 (0.80, 10.31) | 2/3 | 14.06 (1.33, 148.92) |
| Normal | 237/1,164 | 1.00 (ref.) | 126/670 | 1.00 (ref.) |
| Overweight | 361/1,433 | 0.93 (0.74, 1.17) | 308/1,396 | 1.43 (1.09, 1.87) |
| Obesity | 186/608 | 1.12 (0.85, 1.48) | 89/304 | 2.01 (1.40, 2.90) |
| Normal WC | 530/2,395 | 1.00 (ref.) | 533/2,127 | 1.00 (ref.) |
| High WC | 262/824 | 1.31 (1.05, 1.64) | 92/245 | 2.35 (1.69, 3.26) |
| **HUNT3** |  |  |  |  |
| Underweight | 8/16 | 2.89 (0.62, 13.35) | 0/3 | N/A |
| Normal | 182/891 | 1.00 (ref.) | 107/477 | 1.00 (ref.) |
| Overweight | 279/1,326 | 1.04 (0.80, 1.34) | 256/1,370 | 0.95 (0.71, 1.27) |
| Obesity | 200/847 | 1.09 (0.82, 1.46) | 105/487 | 1.20 (0.84, 1.72) |
| Normal WC | 240/1,197 | 1.00 (ref.) | 301/1,616 | 1.00 (ref.) |
| High WC | 431/1,884 | 1.00 (0.80, 1.24) | 166/721 | 1.27 (0.99, 1.62) |
| **HUNT4** |  |  |  |  |
| Underweight | 24/53 | 2.43 (1.22, 4.87) | 4/8 | 3.72 (0.72, 19.28) |
| Normal | 268/1,132 | 1.00 (ref.) | 164/714 | 1.00 (ref.) |
| Overweight | 241/1,324 | 0.69 (0.54, 0.88) | 234/1,410 | 0.75 (0.57, 0.98) |
| Obesity | 153/820 | 0.71 (0.54, 0.94) | 95/506 | 0.92 (0.66, 1.30) |
| Normal WC | 120/909 | 1.00 (ref.) | 173/1,429 | 1.00 (ref.) |
| High WC | 143/1,842 | 0.57 (0.43, 0.75) | 127/920 | 1.27 (0.96, 1.68) |
| *Adjusted for age, educational attainment, marital status, level of physical activity, smoking, symptoms of anxiety or depression, and APOE ɛ4 status. Abbreviations: BMI: body mass index, CI: confidence intervals, HUNT: The Trøndelag Health Study, HUNT1: 1984-86, HUNT2: 1995-97, HUNT3: 2006-08, HUNT4: 2017-19, OR: odds ratio, WC: waist circumference. | | | | |

| **Table S13**. Associations of different combinations of BMI and WC and HUNT2, HUNT3, and HUNT4 with dementia risk vs. no cognitive impairment at HUNT4 in women and men. Performed on multiple imputed data. | | | | |
| --- | --- | --- | --- | --- |
|  | Women | | Men | |
|  | Cases/n | OR (95% CI)* | Cases/n | OR (95% CI)* |
| **HUNT2** |  |  |  |  |
| Normal WC, normal BMI | 233/1,154 | 1.00 (ref.) | 125/669 | 1.00 (ref.) |
| Normal WC, overweight/obesity | 290/1,228 | 0.86 (0.67, 1.08) | 306/1,454 | 1.35 (1.03, 1.77) |
| High WC, normal BMI | 4/10 | 1.06 (0.18, 6.17) | 1/1 | N/A |
| High WC, overweight/obesity | 257/813 | 1.21 (0.93, 1.56) | 91/244 | 2.89 (1.97, 4.23) |
| **HUNT3** |  |  |  |  |
| Normal WC, normal BMI | 138/741 | 1.00 (ref.) | 104/474 | 1.00 (ref.) |
| Normal WC, overweight/obesity | 92/437 | 1.11 (0.78, 1.58) | 195/1,135 | 0.92 (0.68, 1.25) |
| High WC, normal BMI | 43/149 | 1.02 (0.60, 1.76) | 2/2 | N/A |
| High WC, overweight/obesity | 386/1,733 | 1.05 (0.81, 1.37) | 164/719 | 1.19 (0.86, 1.65) |
| **HUNT4** |  |  |  |  |
| Normal WC, normal BMI | 87/738 | 1.00 (ref.) | 87/599 | 1.00 (ref.) |
| Normal WC, overweight/obesity | 19/131 | 1.05 (0.58, 1.90) | 83/823 | 0.67 (0.47, 0.95) |
| High WC, normal BMI | 16/181 | 0.79 (0.45, 1.40) | 2/10 | 0.85 (0.13, 5.53) |
| High WC, overweight/obesity | 126/1,660 | 0.60 (0.43, 0.82) | 125/909 | 1.04 (0.74, 1.46) |
| *Adjusted for age, educational attainment, marital status, level of physical activity, smoking, symptoms of anxiety or depression, and APOE ɛ4 status. Abbreviations: BMI: body mass index, CI: confidence intervals, HUNT: The Trøndelag Health Study, HUNT1: 1984-86, HUNT2: 1995-97, HUNT3: 2006-08, HUNT4: 2017-19, OR: odds ratio, WC: waist circumference. | | | | |

# **References**

[1] Bjerkeset O, Nordahl HM, Mykletun A, Holmen J, Dahl AA. Anxiety and depression following myocardial infarction: gender differences in a 5-year prospective study. J Psychosom Res. 2005;58:153-61. doi:10.1016/j.jpsychores.2004.07.011.

[2] Zigmond AS, Snaith RP. The hospital anxiety and depression scale. Acta Psychiatr Scand. 1983;67:361-70. doi:10.1111/j.1600-0447.1983.tb09716.x.
